# The correlation between ultrasonographic findings and clinical symptoms of pelvic endometriosis

**DOI:** 10.1186/s13104-024-06761-4

**Published:** 2024-04-18

**Authors:** Seyed Reza Saadat Mostafavi, Elham Kor, Seyyed Mohammad Sakhaei, Anis Kor

**Affiliations:** 1 Department of Radiology, Rasoul-E-Akram Hospital, Iran University of Medical Sciences, Tehran, Iran; 2 Department of Radiology, Islamic Azad University-Sari Branch, Sari, Iran

**Keywords:** Endometriosis, Transvaginal ultrasound, Dysmenorrhea

## Abstract

**Objective:**

Considering the importance of endometriosis and its relatively high prevalence among women, this study sought to investigate clinical and Transrectal and transvaginal ultrasounds (TVS) findings of disease.

**Methods:**

This descriptive-analytical study was performed based on medical records of 155 women with endometriosis admitted to Rasool-e Akram Hospital in Tehran for a TVS. All the sonography data and patients’ information were collected into checklists and analyzed in SPSS-25 software (IBM).

**Results:**

The mean age of participants was 32.4 ± 6.1 years, ranging from 18 to 50 years. Endometrioma was prevalent in 129 patients (84.8%). Size of endometrioma (diameter) was more than 3 cm in 79.9% of patients, and 3 cm or fewer in 20.1% of cases. Bladder, intestinal, vaginal, and rectosigmoid involvements with endometriosis implants were observed in 4 (2.6%), 54 (35.5), 3 (0.2%), and 51 (33.5) of patients, respectively. A total of 64.5% of patients were diagnosed with incomplete stenosis of the Douglas pouch and 35.5% had complete stenosis. Deep infiltrating endometriosis (DIE) was less than 1 cm in 20.7%, 1 to 3 cm in 42.3%, and over 3 cm in 37% of patients. The most common manifestations of endometriosis Obliteration of the Douglas pouch, endometrioma, and DIE. In addition, imaging modalities have shown promising results, indicating the necessity to use transvaginal ultrasound as the first line of diagnosis in patients with clinically suspected endometriosis.

**Supplementary Information:**

The online version contains supplementary material available at 10.1186/s13104-024-06761-4.

## Introduction

Endometriosis is a chronic multifocal gynecologic disease, that is common among women of childbearing age. The common morbidity of Endometriosis is chronic pelvic pain and infertility. It may involve invasive fibrotic nodules of the peritoneum, uterine synechiae, and hemorrhagic ovarian cysts. Clinical examinations might contribute to the diagnosis but accurate diagnosis is achieved by imaging techniques [1]. Endometriosis affects 10 to 15% of all women of reproductive age, ranging from 18 to 45 years [2]. It is asymptomatic in the majority of cases but patients may suffer from dysmenorrhea, dyspareunia, and chronic pelvic pain. Endometriosis is common in about 30% of infertile women. Studies have reported an average diagnostic delay of eight years for endometriosis [3]. This delayed diagnosis affects a woman’s quality of life and may result in long-term morbidity, particularly in cases of deep infiltration endometriosis (DIE) of the intestine or vesicoureteral area [4, 5].

Endometriosis is difficult to diagnose by symptoms. It is necessary to measure the risk of endometriosis in patients with suspected history and symptoms by carrying out a physical examination [6]. However, accurate diagnosis of the disease is important for optimum treatment plans [7, 8]. Precise evaluation of pelvic endometriosis needs to be done with endocavitary probes (transvaginal or transrectal) and systematic examination of the uterus, ovaries, adnexa, and the peritoneal covering of the urinary bladder, uterus, Douglas pouch, and rectosigmoid, rectocervical, and rectovaginal regions. A reliable diagnosis of endometriosis can be achieved by laparoscopy and Histopathologic confirmation. However, in patients requiring surgery these techniques are expensive and must be done by experts to avoid misdiagnosis. Additionally, the invasive nature of surgery, makes that less favorable for suspected patients [9, 10]. Ultrasound efficiency in diagnosing endometriosis has been shown in many studies. Transrectal and transvaginal ultrasounds (TVS) have shown promising and potential results in diagnosing non-ovarian endometriosis such as intestine, bladder, and uterosacral ligament involvements. Research has shown the comparable and/or even superior performance of transvaginal ultrasound as compared to MRI [11].

In 2017 research evaluating endometriosis on the uterosacral ligament as a marker of ureteral involvement endometriosis [12], 436 patients diagnosed with DIE underwent TVS endometriosis mapping before laparoscopic surgery for full excision of endometriotic lesions. Of the patients, 23.97% presented with endometriosis nodules. Uterosacral ligament nodule sizes of 1.75 cm and 1.95 cm significantly increased the risk of ureteral involvement, indicating USL nodule size as a key measure for therapeutic planning [9].

Considering the importance of endometriosis and its relatively high prevalence among women as well as the wide range of endometriosis symptoms, we aimed to investigate clinical and TVS findings of disease and correlation between ultrasonography findings and clinical symptoms of pelvic endometriosis.

## Materials and methods

This descriptive-analytical study was performed based on medical records of 155 women with endometriosis referred to Rasool-e Akram Hospital in Tehran for a TVS. Patients suspected of endometriosis enrolled in the study were identified with TVS, and the diagnosis of endometriosis was confirmed by laparoscopic surgery. All ultrasounds were performed by an expert radiologist and all laparoscopies were performed by a gynecologist within two years. Three patients were excluded from the study because of other diagnosis than endometriosis in laparoscopy. All three cases of patients who were excluded from the study had pelvic inflammatory disease. Patients who had previously undergone surgery were not included in the study. All patients enrolled in the study were diagnosed by an expert gynecologist based on the visualization of superficial implants, endometriomas, adhesion distribution, bowel disease and ureteric disease. In doubtful cases, biopsies showing glands and stroma have been the basis of the diagnosis. Data were collected through the researcher-made checklists for assembling patients’ data from medical records including TVS information such as ovarian endometrioma (Appendix 2), endometriosis implants (Appendix 3), stenosis of Douglas pouch, bladder, intestine, rectosigmoid (Appendix 4), and vaginal involvement (Appendix 5), demographic information such as age, height, weight, and body mass index (BMI), and data on the menstrual cycle, the history of delivery and/or infertility, and the disease symptoms.

### Inclusion criteria

- patients with suspected endometriosis with medical records of TVS.

### Exclusion criteria

- other diagnosis than endometriosis in laparoscopic examination.
- loss of data in medical records.

### Symptoms, physical examination, and history of suspected patients for endometriosis [5

- Persistent and/or worsening cyclic or constant pelvic pain.
- Dysmenorrhea (characterized by severe and frequent menstrual cramps and pain during your period).
- Deep dyspareunia (dyspareunia defined as persistent or recurrent genital pain that occurs just before, during, or after intercourse.dyspareunia defined as the extension of pain into the deeper parts of the vagina or lower pelvis).
- Cyclic dyschezia(difficulty in defecating).
- Cyclic dysuria(a symptom of pain and/or burning, stinging, or itching of the urethra or urethral meatus with urination).
- Cyclic catamenial symptoms located in other systems (e.g., lung, skin).
- Infertility.
- Current chronic pelvic pain.
- Dysmenorrhea unresponsive to NSAIDs.
- Positive family history.
- Nodules in cul de sac in physical exam (PE).
- Retroverted uterus in PE.
- Mass consistent with endometriosis in PE.
- Obvious endometrioma that is external (seen on speculum or skin).

All patients had a dynamic ultrasonography with four steps [13]:

#### First step

Routine evaluation of uterus and adnexa (+ sonographic signs of adenomyosis/presence or absence of endometrioma [14].

#### Second step

Evaluation of transvaginal sonographic ‘soft markers’ (i.e. site-specific tenderness and ovarian mobility [15].

#### Third step

Assessment of the status of the pouch of Douglas using real-time ultrasound-based ‘sliding sign‘ [15].

#### Fourth step

Assessment for DIE nodules in anterior and posterior compartments [16].

### Ultrasound findings

1. Endometrioma: Characteristic US features of an endometrioma Include a Round, homogeneous, hypoechoic, low-level echo cyst, without internal vascularity and no or poor vascularization of the capsule and septa. Appendix 1.
2. DIE: endometriosis nodule in the uterosacral ligament, intestine, bladder, rectum, rectosigmoid, and rectocervical area with involvement of the muscularis propria layer. Appendices 2, 3.
3. Posterior DIE: nodule in Rectovaginal and rectocervical septum and uterosacral ligament. Appendix 4.
4. Douglas Stenosis: adhesion of the rectosigmoid junction or anterior rectal wall to the cervix.
5. Presence of Adenomyosis: presence of ectopic endometrial tissue in the myometrium. Appendix 5.

### Endometriosis stages [17

#### Stage 1: Minimal

: There are a few isolated and superficial implants of endometrial-like tissue.

#### Stage 2: Mild

Unilateral or bilateral endometriomas with a diameter less than 3 cm; ovaries in normal sites, mobile, and not adherent to the uterus and surrounding tissues.

#### Stage 3: Moderate

Unilateral or bilateral endometriomas with a diameter greater than 3 cm; at least one ovary in normal site, mobile, and not adherent to the uterus and surrounding tissues, or one ovary adherent only to the uterus or broad ligament.

#### Stage 4: Severe

unilateral or bilateral endometriomas with a diameter greater than 3 cm; ovaries in abnormal sites (prolapsed in the pouch of Douglas or dislocated posteriorly, anteriorly, or superiorly to the uterus) and adherent to the uterus and surrounding tissues; presence of pelvic adhesions or endometriotic nodules.

All data were organized in Excel and then analyzed in SPSS-25 software (IBM) using descriptive statistics (mean, SD, and rate of change for quantitative variables, as well as frequency and percentage of prevalence for qualitative data, chi-square (X^2^) test was applied to assess the relationship between clinical and ultrasound findings, Multiple logistic regression analysis was used to estimate the odds ratio (OR) with a 95% confidence interval (CI) for the risk factors and Endometriosis. In all tests, the confidence interval was considered 95%, and *P* value < 0.05 was significant.

## Results

Patients were aged from 18 to 50 (32.4 ± 6.1). A total number of 54 (35.5%) were less than 30 years old, 31 (20.4%) were between 30 and 33, and 67 (44.1%) of patients were over 33. The mean height, weight, and body mass index (BMI) of patients were 164 ± 5.9, 63.2 ± 10.2, and 23.4 ± 3.7, respectively.

Duration of infertility ranged from 12 to 240 months (59.1 ± 56.9). The mean age at onset of menarche and days of menstruation cycle was 13.1 ± 1.5, and 12.9 ± 7.2, respectively.

A total number of 50 (32.9%) patients had hypermenorrhea, 28 (18.4%) Polymenorrhea, and 10 (6.6%) Oligomenorrhea. The most common clinical manifestations of endometriosis were dysmenorrhea (95.4%), abdominal pain (89.6%), chronic fatigue (65.1%), chronic pelvic pain (47.1%), and dyspareunia (47.1%).

Endometrioma was reported in 129 (84.8%), of which 78 (60.8%) were unilateral and 51 (39.2%) were bilateral endometrioma. Endometrioma diameter was more than 3 cm in 79.9%, and 3 cm or less in 20.1% of patients.

Bladder, intestinal, vaginal, and rectosigmoid involvements with endometriosis implants were observed in 4 (2.6%), 54 (35.5), 3 (0.2%), and 51 (33.5) of patients, respectively.

Stenosis of Douglas pouch was present in 131 (86.2%) of patients, of which 54 (35.5%), and 77 (64.5%) were complete and incomplete stenosis, respectively. DIE was reported in 111 (73%) of the studied patients. The size of DIE implants was less than 1 cm in 20.7%, 1 to 3 cm in 42.3%, and more than 3 cm in 37% of cases.

The correlation between TVS findings and the clinical aspect of the disease is shown in Tables 1, 2 and 3. Dysmenorrhea severity was related to intestine involvement (*p* = 0.035), DIE implant size (0.016), and Posterior DIE implant size (0.013). Dyspareunia severity was related to Douglas Stenosis (*p* = 0.044). In addition, Chronic Pelvic Pain Severity is associated with Endometrioma (*p* = 0.024), DIE implant size (0.039), and Posterior DIE implant size (0.034). The results of the logistic regression test for dysmenorrhea, dysmenorrhea severity, dyspareunia, dyspareunia severity as well and chronic pelvic pain showed that Dyspareunia was related to the stage of endometriosis, and the odds ratio of endometriosis in the absence of dyspareunia was 0.24. (Appendix 1)

**Table 1:** TVS findings in different severity of dysmenorrhea in endometriosis patients

Dysmenorrhea Severity		Minimal	Mild	Moderate	Severe	*p*-value
		*n* (%)	*n* (%)	*n* (%)	*n* (%)	
Endometrioma	None	2 (9.5)	5 (11.1)	10 (17.2)	7 (25.0)	0.303
	< 3 cm	7 (33.3)	6 (13.3)	11 (19.0)	4 (14.3)	
	> 3 cm	12 (57.1)	34 (75.6)	37 (63.8)	17 (60.7)	
superficial peritoneal implants	None	20 (95.2)	40 (88.9)	55 (94.8)	27 (96.4)	0.403
	< 3 cm	1 (4.8)	3 (6.7)	0 (0.0)	1 (3.6)	
	> 3 cm	0 (0.0)	2 (4.4)	3 (5.2)	0 (0.0)	
Endometrioma	Yes	19 (90.5)	40 (88.9)	48 (82.8)	21 (75.0)	0.356
	No	2 (9.5)	5 (11.1)	10 (17.2)	7 (25.0)	
Type Of Endometrioma	Unilateral	40 (88.9)	48 (82.8)	21 (75.0)	128 (84.2)	0.171
	Bilateral	5 (11.1)	10 (17.2)	7 (25.0)	24 (15.8)	
Douglas Stenosis	Complete	22 (55.0)	31 (64.6)	10 (47.6)	78 (60.9)	0.104
	Partial	18 (45.0)	17 (35.4)	11 (52.4)	50 (39.1)	
	Absent	40 (100.0)	48 (100.0)	21 (100.0)	128 (100.0)	
Bladder Involvement	Yes	11 (24.4)	17 (29.3)	15 (53.6)	53 (34.9)	0.134
	No	27 (60.0)	33 (56.9)	8 (28.6)	76 (50.0)	
Intestine Involvement	Yes	1 (2.2)	0 (0.0)	1 (3.6)	4 (2.6)	*0.035*
	No	44 (97.8)	58 (100.0)	27 (96.4)	148 (97.4)	
Sub peritoneal expansion	Sub peritoneal only	14 (66.7)	0 (62.2)	44 (75.9)	12 (42.9)	0.059
	Rectal	7 (33.3)	16 (35.6)	12 (20.7)	16 (57.1)	
	Vaginal	0 (0.0)	1 (2.2)	0 (0.0)	0 (0.0)	
	Both Rectal and Vaginal	0 (0.0)	0 (0.0)	2 (3.4)	0 (0.0)	
DIE Implants	None	8 (38.1)	0 (13.3)	22 (37.9)	6 (21.4)	*0.016*
	< 1 cm	2 (9.5)	9 (20.0)	11 (19.0)	0 (0.0)	
	1–3 cm	5 (23.8)	19 (42.2)	13 (22.4)	10 (35.7)	
	> 3 cm	6 (28.6)	11 (24.4)	12 (20.7)	12 (42.9)	
Posterior DIE Implants	None	9 (42.9)	0 (15.6)	22 (37.9)	6 (21.4)	*0.013*
	< 1 cm	2 (9.5)	9 (20.0)	11 (19.0)	0 (0.0)	
	1–3 cm	4 (19.0)	19 (42.2)	13 (22.4)	10 (35.7)	
	> 3 cm	6 (28.6)	10 (22.2)	12 (20.7)	12 (42.9)	
Endometriosis staging	minimal	0 (0.0)	1 (8.3)	2 (16.7)	9 (75)	*0.61*
	mild	0 (0.0)	0 (0.0)	3 (30.0)	7 (70)	
	moderate	0 (0.0)	9 (15.8)	18 (31.6)	30 (52.6)	
	severe	4 (5.5)	7 (9.6)	22 (30.1)	40 (54.8)	

**Table 2:** TVS findings in different severity of dyspareunia in endometriosis patients

Dyspareunia Severity		Minimal	Mild	Moderate	Severe	*p*-value
		*n* (%)	*n* (%)	*n* (%)	*n* (%)	
Endometrioma	None	12 (12.0)	8 (25.8)	3 (18.8)	1 (20.0)	0.442
	< 3 cm	19 (19.0)	7 (22.6)	2 (12.5)	0 (0.0)	
	> 3 cm	69 (69.0)	16 (51.6)	11 (68.8)	4 (80.0)	
superficial peritoneal implants	None	94 (94.0)	27 (87.1)	16 (100.0)	5 (100.0)	0.410
	< 3 cm	2 (2.0)	3 (9.7)	0 (0.0)	0 (0.0)	
	> 3 cm	4 (4.0)	1 (3.2)	0 (0.0)	0 (0.0)	
Endometrioma	Yes	88 (88.0)	23 (74.2)	13 (81.3)	4 (80.0)	0.309
	No	12 (12.0)	8 (25.8)	3 (18.8)	1 (20.0)	
Type Of Endometrioma	Unilateral	23 (74.2)	13 (81.3)	4 (80.0)	128 (84.2)	0.166
	Bilateral	8 (25.8)	3 (18.8)	1 (20.0)	24 (15.8)	
Douglas Stenosis	Complete	11 (47.8)	11 (84.6)	3 (75.0)	78 (60.9)	*0.044*
	Partial	12 (52.2)	2 (15.4)	1 (25.0)	50 (39.1)	
	Absent	23 (100.0)	13 (100.0)	4 (100.0)	128 (100.0)	
Bladder Involvement	Yes	18 (58.1)	7 (43.8)	1 (20.0)	53 (34.9)	0.882
	No	8 (25.8)	8 (50.0)	3 (60.0)	76 (50.0)	
Intestine Involvement	Yes	1 (3.2)	0 (0.0)	0 (0.0)	4 (2.6)	0.463
	No	30 (96.8)	16 (100.0)	5 (100.0)	148 (97.4)	
Sub peritoneal expansion	Sub peritoneal only	66 (66.0)	0 (54.8)	11 (68.8)	4 (80.0)	0.230
	Rectal	33 (33.0)	13 (41.9)	4 (25.0)	1 (20.0)	
	Vaginal	0 (0.0)	0 (0.0)	1 (6.3)	0 (0.0)	
	Both Rectal and Vaginal	1 (1.0)	1 (3.2)	0 (0.0)	0 (0.0)	
DIE Implants	None	28 (28.0)	0 (25.8)	4 (25.0)	2 (40.0)	0.153
	< 1 cm	19 (19.0)	1 (3.2)	1 (6.3)	1 (20.0)	
	1–3 cm	28 (28.0)	9 (29.0)	9 (56.3)	1 (20.0)	
	> 3 cm	25 (25.0)	13 (41.9)	2 (12.5)	1 (20.0)	
Posterior DIE Implants	None	30 (30.0)	0 (25.8)	4 (25.0)	2 (40.0)	0.128
	< 1 cm	19 (19.0)	1 (3.2)	1 (6.3)	1 (20.0)	
	1–3 cm	27 (27.0)	9 (29.0)	9 (56.3)	1 (20.0)	
	> 3 cm	24 (24.0)	13 (41.9)	2 (12.5)	1 (20.0)	
Endometriosis staging	minimal	5 (45.5)	0 (0.0)	4 (36.4)	2 (18.2)	0.48
	mild	4 (44.4)	0 (0.0)	3 (33.3)	2 (22.2)	
	moderate	21 (47.7)	8 (18.2)	7 (15.9)	8 (18.2)	
	severe	20 (34.5)	12 (20.7)	17 (29.3)	9 (15.5)	

**Table 3:** TVS findings in different severity of chronic pelvic pain in endometriosis patients

Chronic Pelvic Pain Severity		Minimal	Mild	Moderate	Severe	*P*-value
		*n* (%)	*n* (%)	*n* (%)	*n* (%)	
Endometrioma	None	13 (16.5)	0 (0.0)	8 (27.6)	3 (23.1)	0.142
	< 3 cm	15 (19.0)	6 (19.4)	5 (17.2)	2 (15.4)	
	> 3 cm	51 (64.6)	25 (80.6)	16 (55.2)	8 (61.5)	
superficial peritoneal implants	None	74 (93.7)	27 (87.1)	29 (100.0)	12 (92.3)	0.284
	< 3 cm	2 (2.5)	3 (9.7)	0 (0.0)	0 (0.0)	
	> 3 cm	3 (3.8)	1 (3.2)	0 (0.0)	1 (7.7)	
Endometrioma	Yes	66 (83.5)	31 (100.0)	21 (72.4)	10 (76.9)	*0.024*
	No	13 (16.5)	0 (0.0)	8 (27.6)	3 (23.1)	
Type Of Endometrioma	Unilateral	31 (100.0)	21 (72.4)	10 (76.9)	128 (84.2)	0.329
	Bilateral	0 (0.0)	8 (27.6)	3 (23.1)	24 (15.8)	
Douglas Stenosis	Complete	15 (48.4)	12 (57.1)	7 (70.0)	78 (60.9)	0.421
	Partial	16 (51.6)	9 (42.9)	3 (30.0)	50 (39.1)	
	Absent	31 (100.0)	21 (100.0)	10 (100.0)	128 (100.0)	
Bladder Involvement	Yes	15 (48.4)	10 (34.5)	6 (46.2)	53 (34.9)	0.407
	No	14 (45.2)	14 (48.3)	5 (38.5)	76 (50.0)	
Intestine Involvement	Yes	2 (6.5)	0 (0.0)	0 (0.0)	4 (2.6)	0.294
	No	29 (93.5)	29 (100.0)	13 (100.0)	148 (97.4)	
Sub peritoneal expansion	Sub peritoneal only	52 (65.8)	0 (58.1)	18 (62.1)	10 (76.9)	0.923
	Rectal	25 (31.6)	12 (38.7)	11 (37.9)	3 (23.1)	
	Vaginal	1 (1.3)	0 (0.0)	0 (0.0)	0 (0.0)	
	Both Rectal and Vaginal	1 (1.3)	1 (3.2)	0 (0.0)	0 (0.0)	
DIE Implants	None	21 (26.6)	0 (16.1)	11 (37.9)	5 (38.5)	*0.039*
	< 1 cm	15 (19.0)	6 (19.4)	0 (0.0)	1 (7.7)	
	1–3 cm	24 (30.4)	6 (19.4)	13 (44.8)	4 (30.8)	
	> 3 cm	19 (24.1)	14 (45.2)	5 (17.2)	3 (23.1)	
Posterior DIE Implants	None	22 (27.8)	0 (19.4)	11 (37.9)	5 (38.5)	*0.034*
	< 1 cm	15 (19.0)	6 (19.4)	0 (0.0)	1 (7.7)	
	1–3 cm	24 (30.4)	5 (16.1)	13 (44.8)	4 (30.8)	
	> 3 cm	18 (22.8)	14 (45.2)	5 (17.2)	3 (23.1)	
Endometriosis staging	minimal	7 (58.3)	0 (0.0)	2 (16.7)	3 (25.0)	*0.02*
	mild	5 (50.0)	0 (0.0)	5 (50.0)	0 (0.0)	
	moderate	35 (60.3)	9 (15.5)	10 (17.2)	4 (6.9)	
	severe	32 (44.7)	22 (30.6)	13 (18.1)	5 (6.9)	

## Discussion

Dysmenorrhea, abdominal pain, chronic fatigue, chronic pelvic pain, and dyspareunia were the most common symptoms in this study. Reid et al. [12] evaluated the optimal method of sonography for deep rectal and rectosigmoid involvement in endometriosis patients. 410 patients with endometriosis were studied, the most common symptoms of which included dysmenorrhea, dyspareunia, and dyschezia. In the study by Chapron et al. [18], the most common symptoms included dysmenorrhea, chronic pelvic pain, dyspareunia, and intestinal and bladder symptoms. Roughly one-third of the patients in our study had irregular menstrual cycles.

In our study, the overall prevalence of endometrioma was 84.8%, of which 60.8% were unilateral and 39.2% were bilateral. The endometrioma cyst size (diameter) was more than 3 cm in about 80% of patients. The most common sites involved with endometriosis were intestine, rectosigmoid, bladder, and vagina.There is no relationship between the size of the endometrioma and the severity of dyspareunia. Reid et al. reported the highest involvement in the rectum, rectosigmoid, vagina, and uterosacral ligament [12]. Moreover, unilateral and bilateral endometriomas were observed in two-thirds and one-third of patients, which are also in agreement with the results of the present study.

The mean (± SD) of the age of patients in the present study was 32.4 ± 6.1, in agreement with previous research by Ghatresamani et al. [11], which studied 60 patients with suspected endometriosis by transvaginal ultrasound and laparoscopic assessments, they reported the mean age of 31.1 ± 4.97 years for patients. Also, In the study of Holland et al. [1], the mean age of the patients was 35 years. Studies by Kennedy, Poindexter, and Kirshon [19, 20] have shown that aging affects endometriosis so that as a person ages, she experiences more menstrual cycles and may have longer periods with more bleeding. This increases the chance of retrograde menstruation. Aging affects the immune system which may facilitate migration of endometrial cells, it also increases the chance of development of hormonal disorders and uterine abnormalities that lead to irregular mensuration. These may result in retrograde menstruation and progression of endometriosis, which is consistent with the findings of the present study, with more than two-thirds of patients over 30 years of age. Timely diagnosis and identifying the disease as soon as possible and starting the treatment may reduce pain, prevent the progression of the disease and thus preserve fertility [21]. 

Deep infiltrative endometriosis (DIE) is defined as endometriotic tissue found more than 5 mm below the peritoneal surface. DIE might also involve the pouch of Douglas, the vesicouterine pouch, and other pelvic areas [11]. The majority of involvements were observed in the intestine and bladder, similar to the study by Ghatresamani et al. [11] in which most severe involvements were reported in ligaments, intestine, and bladder.

In this study patients with dyspareunia, are five times more likely to have endometriosis than patients without dyspareunia. In previous studies, many attempts have been made to clarify the relationship between the type and location of lesions and the stage of the disease with the severity and symptoms of the disease, but there has been no consensus on the results [22].

## Conclusion

The most obvious manifestations of endometriosis were dysmenorrhea, abdominal pain, chronic fatigue, chronic pelvic pain, and dyspareunia. Early diagnosis and proper treatment of endometriosis can prevent serious morbidities such as infertility and decreased quality of life. Our results indicate the importance of imaging modalities in endometriosis, stenosis of Douglas pouch, endometrioma, and DIE were the most common manifestations in TVS. Results indicate the necessity of TVS as the first-line imaging technique in the diagnosis of endometriosis for clinically suspected patients.

### Limitations

The sample size was short and the study is done in a single center which is better to be performed in some centers with larger sample sizes.

### Electronic supplementary material

Below is the link to the electronic supplementary material.

Supplementary Material 1

## Data Availability

All of the data related to this study are mentioned in this article.

## Abbreviations

TVS: Transrectal and transvaginal ultrasounds
DIE: Deep infiltrating endometriosis
BMI: Body mass index
