# Supplementary material for: The correlation between ultrasonographic findings and clinical symptoms of pelvic endometriosis
Source: BMC Res Notes. 2024 Apr 18;17:108. doi: 10.1186/s13104-024-06761-4 (PMC11027415; doi:10.1186/s13104-024-06761-4)
Supplement: Supplementary file 1 — Supplementary Material 1 [file 13104_2024_6761_MOESM1_ESM.docx]

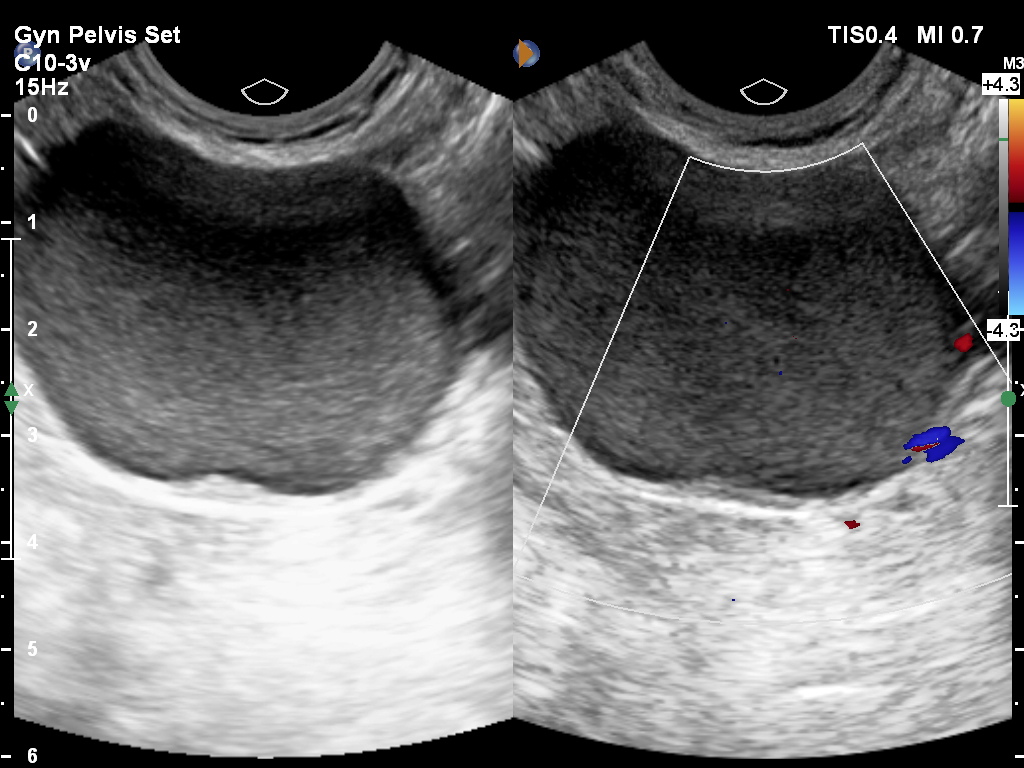


**A**  **B**

**Appendix 1.** Typical appearance of ovarian endometriomas in a 32-year-old; Woman with chronic pelvic pain and a laparoscopically confirmed endometrioma. A) Typical appearance of an ovarian endometrioma in gray scale: a unilocular cyst containing homogeneous low-level echoes, B) No internal vascularity at color Doppler US


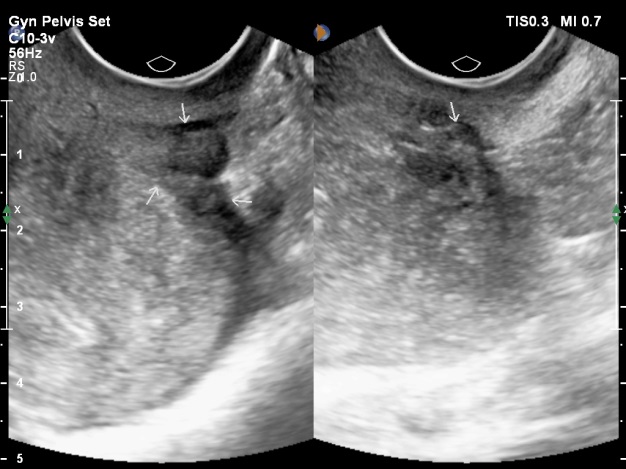

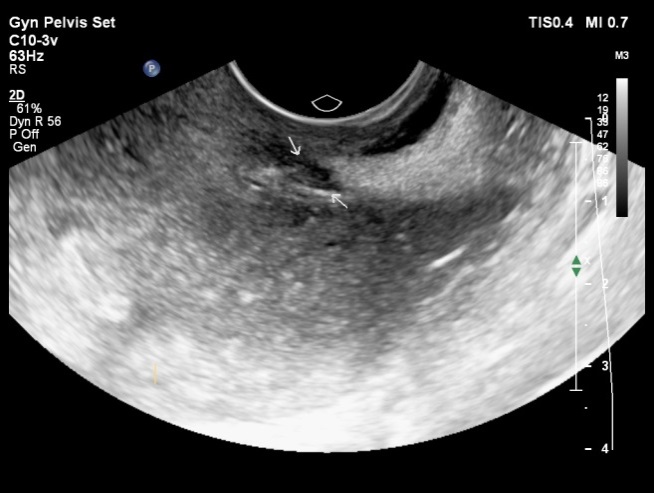


A B

**Appendix 2.** Large USL DIE in two women. A) Sagittal and transverse gray-scale TVS image in a 26-year-old woman with severe dysmenorrhea and chronic pelvic pain and a history of endometriosis show thickening of right USL and a round hypoechoic nodule within USL (arrow), B) Transverse gray-scale TVS image in a 33-year-old woman with a history of severe dyspareunia and stage III endometriosis were confirmed at laparoscopy shows severe thickening of the USL associated hypoechoic nodule (arrows)


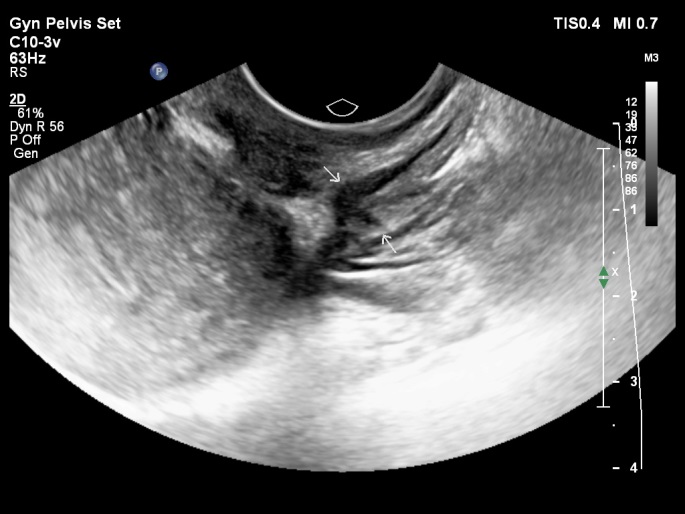

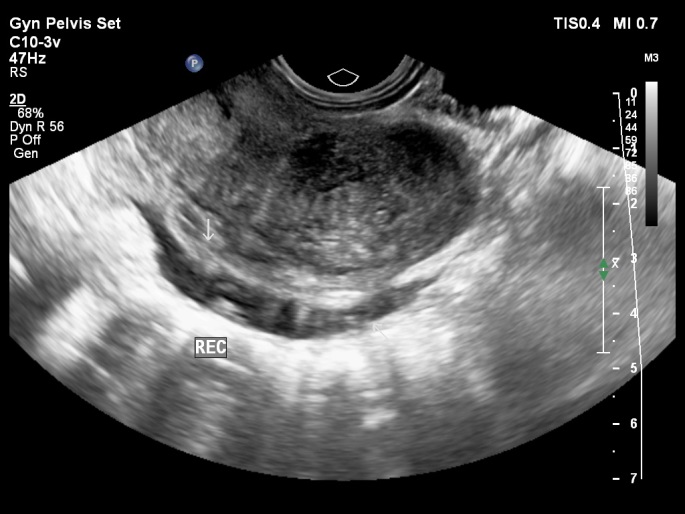


A B


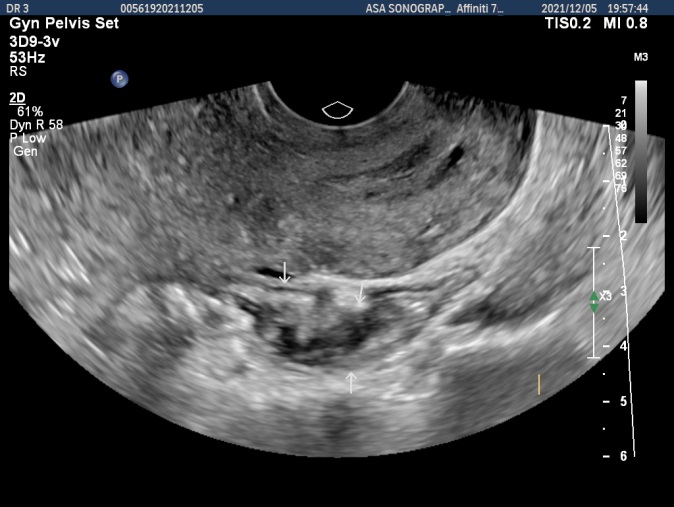

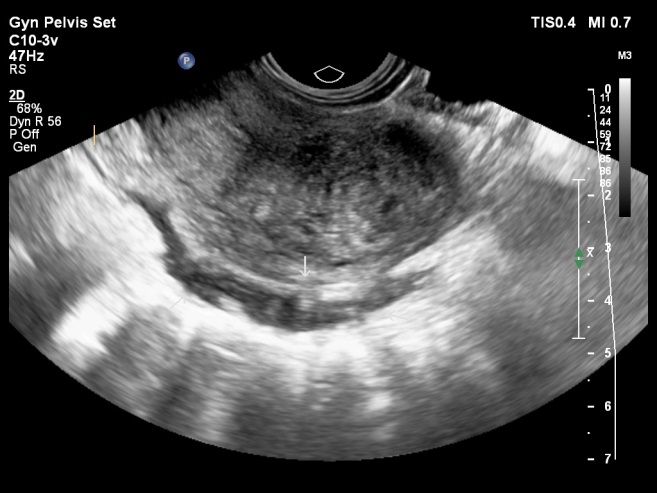


C D

**Appendix 3**. Sonographic appearances of intestine DIE in four women with history of severe endometriosis; (A) Sagittal gray-scale TVS image in a 30-year-old woman shows a hypoechoic nodule in rectum (arrow) with involvement of muscularis propria layer, (B) Sagittal gray-scale TVS image in a 32-year-old woman shows an irregularly shaped nodule (arrow) with prominent spikes towards the bowel lumen (Indian headdress sign), (C) Sagittal gray-scale TVS images in a 28-year-old woman show an irregular hypoechoic nodule (arrow) in rectocervical area, (D) Sagittal gray-scale Transrectal images in a 17-year-old virgin girl show a hypoechoic nodule (arrow) in rectosigmoid area


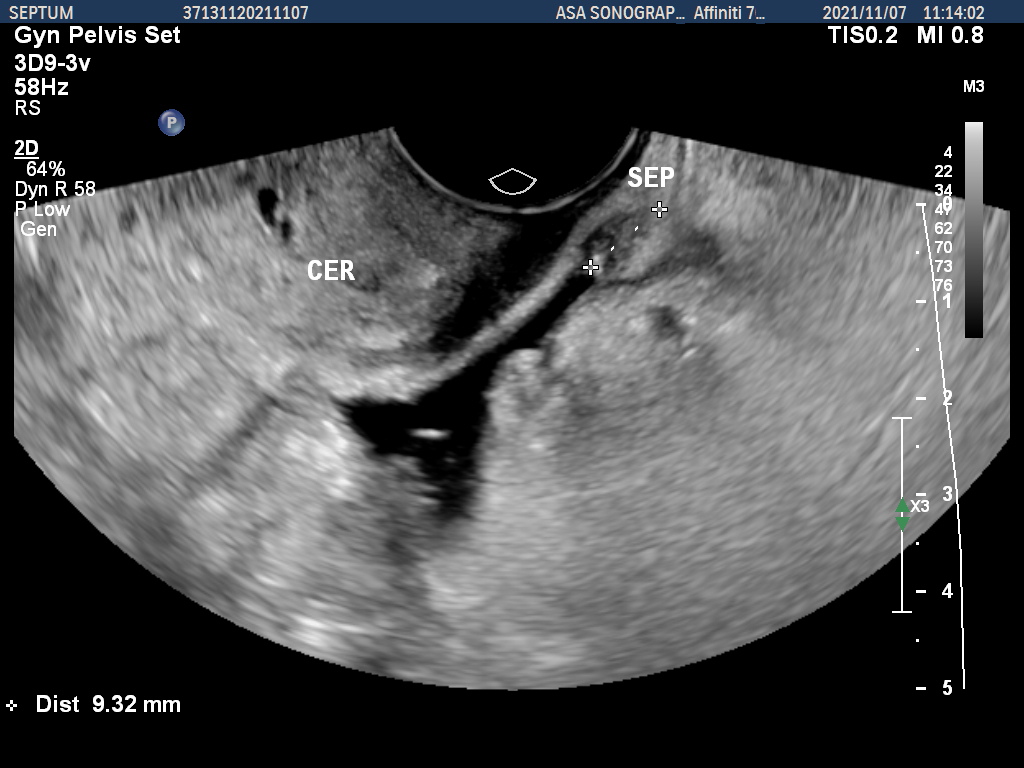


**Appendix 4.** Rectovaginal DIE in a 33-year-old woman with a history of stage IV endometriosis. Sagittal gray-scale TVS images show hypoechoic nodule in Rectovaginal septum.


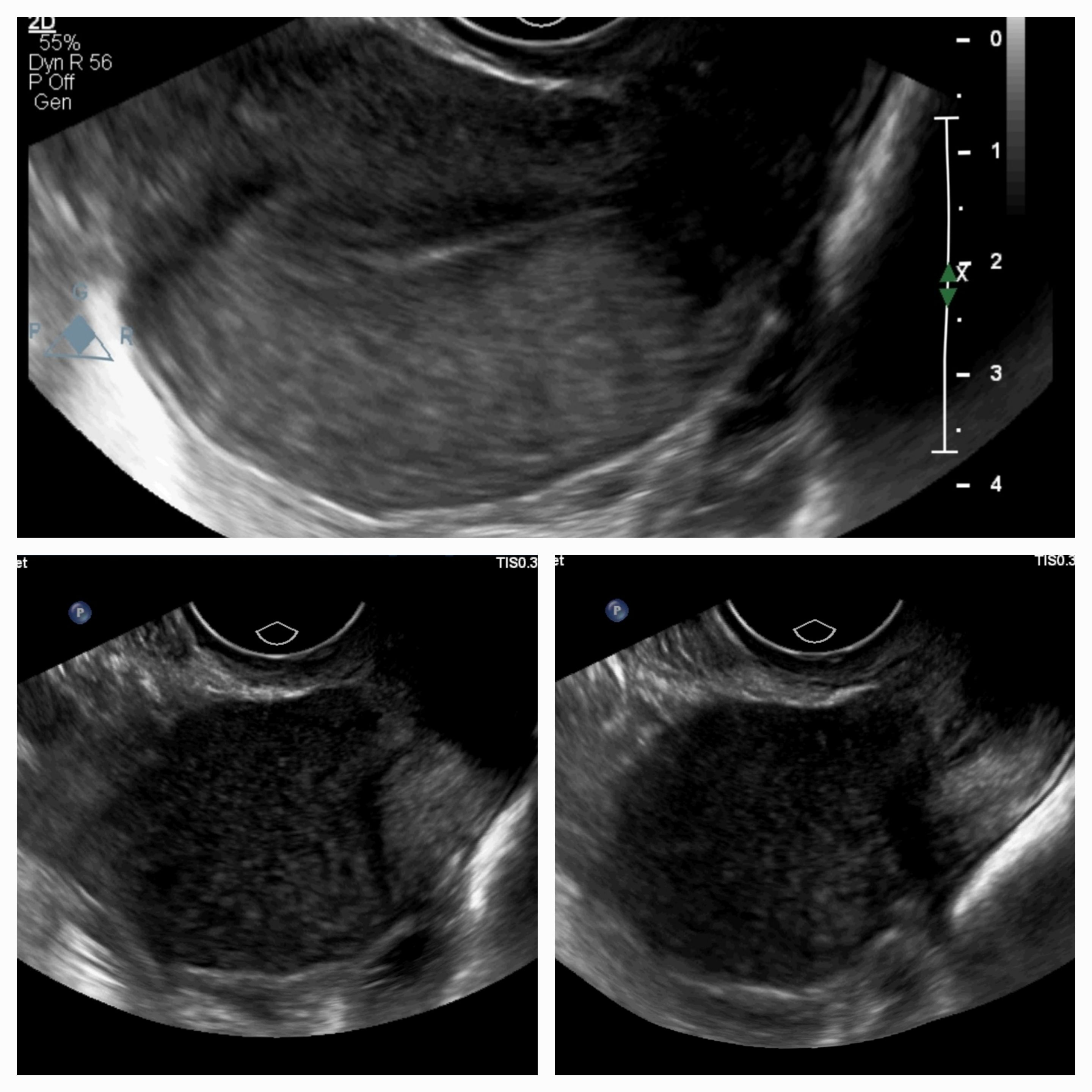


**Appendix 5.** Adenomyosis in a 44-year-old woman with diagnosis of pelvic endometriosis (confirmed bylaparoscopy). Sagittal gray-scale TVS images show large uterus with myometrial thickening and a heterogeneous myometrium with irregular endometrial–myometrial junctio) the upper image) with subendometrial echogenic nodules extending from the endometrium and into the inner myometrium) the lower images)
